# Making friends: active selection of symbionts and rejection of pathogens by the neonatal immune system

**DOI:** 10.3389/fimmu.2023.1287518

**Published:** 2023-11-20

**Authors:** Colleen J. Sedney, Eric T. Harvill

**Affiliations:** Department of Infectious Diseases, College of Veterinary Medicine, University of Georgia, Athens, GA, United States

**Keywords:** neonatal, immunity, microbiota, immune system development, neonatal immune system

## Abstract

The neonatal immune system is generally viewed as deficient compared to adults, often attributed to its incomplete development. This view is reinforced by the extraordinary sensitivity and susceptibility of neonates to certain pathogens. Examination of the basis for this susceptibility has characterized neonatal immunity as skewed strongly toward anti-inflammatory responses, which are interpreted as the lack of full development of the strong inflammatory responses observed in adults. Here we examine the alternative explanation that neonatal immune responses are generally complete in healthy newborns but evolved and adapted to very different functions than adult immunity. Adult immunity is primarily aimed at controlling pathogens that invade the holobiont, with substantial competition and protection conferred by resident microbiota. Rather than simply repelling new invaders, the immediate and critical challenge of the neonatal immune system during the sudden transition from near sterility to microbe-rich world is the assimilation of a complex microbiota to generate a stable and healthy holobiont. This alternative view of the role of the neonatal immune system both explains its strong anti-inflammatory bias and provides a different perspective on its other unique aspects. Here we discuss recent work exploring the initial contact of newborns with microbes and their interactions with neonatal immune responses, contrasting these alternative perspectives. Understanding how the need to rapidly acquire a highly complex and rich microbiota of commensals affects interactions between the neonatal immune system and both commensals and pathogens will allow more targeted and effective collaboration with this system to quickly achieve a more disease-resistant holobiont.

## 1 Introduction

Neonates/infants and adults have vastly different immune systems, with a growing list of profound differences in immune cells and their functions that leave newborns highly susceptible to certain pathogens. This apparent failure, and the later transition to a more adult-like immune system, support a common view that the neonatal immune system (NIS) is an incompletely developed version of the adult immune system. However, newer work suggests that rather than being simply deficient, the NIS has different, and in some cases more effective, responses than the adult immune system (AIS), with the potential for vigorous responses being more tightly controlled (1, 2). These neonatal-specific responses, believed to prevent damaging inflammation, appear to contribute to the increased susceptibility of neonates to a subset of pathogens.

Contrary to the current dogma, neonates are not highly susceptible to all pathogens; the NIS can protect the host against most pathogens and therefore must possess some competent, albeit different, immune defenses (3). To understand the important differences between the NIS and AIS, it may be useful to consider the very different challenges that are unique to the NIS. Specifically, the NIS has an additional responsibility beyond simply identifying and rejecting pathogens, which is the acquisition of a multitude of commensals that make up the complete microbiota. This is done at several body surfaces simultaneously, beginning immediately at birth and evolving rapidly based on the status of the microbiota and the associated host tissues. Performing this critical function while simultaneously surveilling for pathogens is a challenge particular to the NIS, which may explain some or all of its differences from the AIS (4, 5). This viewpoint may also contribute a new perspective on why the NIS appears to be less effective against particular pathogens.

Others have proposed that the NIS must be less inflammatory to deal with the onslaught of new organisms at birth, taking a more passive approach that avoids unnecessary inflammatory responses (6). This perspective remains firmly focused on the role of the immune system as being control/elimination of pathogens, which is understandable considering the profound burden of infectious disease. However, we and independently McFall-Ngai, have previously proposed that the immune system may be viewed more broadly as a system for managing the incredibly important mammalian microbiota that, together with the host, make up a healthy holobiont (4, 5). Our novel perspective here is an extension of that view, being that the NIS is not simply avoiding unnecessary damaging responses to the many new microbes and antigens encountered after birth, but has as its primary function the healthy assimilation of a complete microbiota, and is actively involved in that process. The critical transition from near sterile fetus *in utero* to a complete holobiont, the aggregate of host and its microbiota, occurs in the days and weeks after birth, requiring an immune system that functions very differently from that of an adult holobiont with a complete, stable and healthy microbiota. Here we will contrast the common view of the NIS as an incompletely developed AIS, with this alternative, and not necessarily mutually exclusive, view of the NIS as a highly evolved system for actively acquiring the complex microbiota to achieve a healthy holobiont.

**Figure 1:**
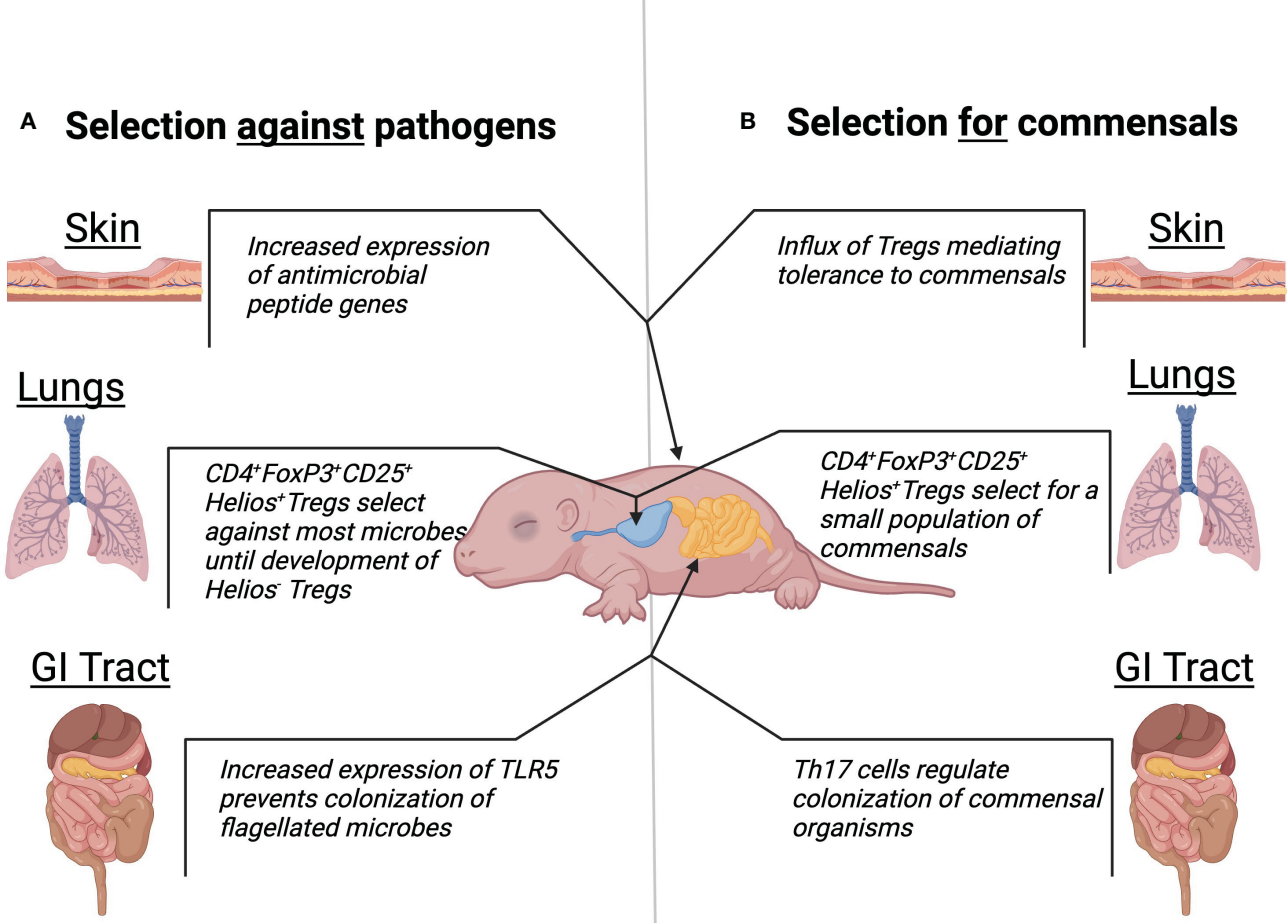
Traditional and alternative view of neonatal microbiota development. Mechanisms at the skin, lungs, and GI tract body sights which suggest a role of the neonatal microbiota to select against pathogenic colonization **(A)**. Mechanisms at the skin, lungs, and GI tract body sights which suggest a role of the neonatal microbiota to select for colonization of healthy commensals **(B)**.

We review a subset of recent experimental animal studies of early neonatal interactions with microbes that mostly focus on limitations and weaknesses of the NIS. We reinterpret these findings in light of the view that the NIS at birth is well evolved for the unique challenges of acquiring a healthy microbiota (**Figure 1**). Observations such as the relative tolerance to many MAMPs and PAMPs, which could be considered a weakness in defense against pathogens, are reconsidered as critical for the healthy assimilation of microbiota. One key concept that derives from this perspective is that the NIS may simultaneously select for harmless and potentially co-evolved commensal organisms and against pathogens to establish a protective microbiota. Additionally, apparent failures of the NIS to deal with some pathogens are discussed in the context of the prevailing need to rapidly acquire microbiota. The aim of this perspective is to reinterpret recent data concerning the NIS as a highly sophisticated system which efficiently meets the critical and urgent need for the host to acquire a healthy microbiota, with the hope that this perspective leads to reinterpretation of some studies and more new considerations and experiments.

## 2 Organ systems

### 2.1 Skin

The first area to be exposed to the outside world, and thus develop a microbiota, is the skin. It is also considered to be the first line of defense against all future pathogens, making acquiring healthy skin microbiota crucial. Importantly, commensals of the skin can cause disease in other host sites, such as the nasal cavity, lungs, or GI tract, therefore the NIS must allow for their growth on the skin while preventing colonization/growth in other areas. The skin of newborn mice has been shown to have increased production of antimicrobial peptides (AMP) compared to older mice, primarily of the cathelicidin and B-defensin families; expression of mouse cathelicidin (CRAMP) was 10- to 100-fold higher in perinatal skin than adult murine skin (7). Importantly, commensals of the skin, such as *S. epidermidis*, induce the production of, and are thus resistant to, AMPs which kill pathogenic group B *Streptococcus* (GBS) and *S. aureus* (8, 9). This suggests a highly evolved synergistic relationship between the neonatal immune system and skin commensals which promotes their colonization and a protective response against pathogenic microbes prior to cellular intervention (7).

While the roles of cellular immune components in the skin of neonates are not as well understood as that of other body areas, several studies have identified important contributions of cell-mediated regulation of skin inflammation and bacterial colonization. Regulatory T cells (Tregs) that are uniquely prevalent in the NIS contribute to tightly controlling inflammatory responses. A recent study demonstrated that neonates have a crucial window of colonization that is characterized by an abrupt influx of highly activated Tregs (10). These Tregs are important for the establishment of immune tolerance to commensal microorganisms and inhibition of these cells abrogated tolerance and resulted in disease (10). Neonatal mice also have a transient reduction of Tregs in the skin which causes an inflammation-mediated dysregulation of subcutaneous tissue in skin, which primes the skin for altered reparative responses to wounding (11). This suggests that populations of Tregs in the skin shift in response to bacterial colonization. While these studies suggest a role for Tregs in regulating inflammation, they also demonstrate a role in selecting for healthy commensal skin microorganisms, with knock on effects when this role is disrupted. Leech et al., 2019 demonstrated that neonatal exposure to the commensal *S. epidermidis* facilitates specific Treg tolerance, while a toxin produced by the pathogenic species *S. aureus* mediates influxes of effector T cells and subsequent inflammation (5). Tregs in adults are known to have anti-pathogen mechanisms, however this has not been investigated in neonatal models. Considering a critical role in mediating microbiota acquisition suggests that in addition to selecting against pathogens, the NIS may select for healthy, co-evolved commensals that have acquired resistance to skin antimicrobials and may interact with Tregs to reduce inflammation. Further studies contrasting the different interactions of commensals and pathogens with neonatal Tregs might reveal important mechanisms by which they are distinguished and encouraged or discouraged.

### 2.2 Lungs

The lungs are known to be one of the most disease-susceptible organs in adults and newborns. To function properly, their vast surface area must remain nearly sterile despite the repeated introduction of microbe-rich air. Inflammatory responses can impede respiratory function and may be more disastrous than the infection itself, particularly in infants. While a protective microbiota is an important factor for preventing pathogenic colonization, its establishment may be particularly challenging. This suggests that a complex and delicate system must exist which allows the deposition and colonization of commensal organisms, while preventing pathogen colonization with minimal inflammation. Similar to the skin, neonatal mice have high amounts of CD4^+^FoxP3^+^CD25^+^Helios^+^ Tregs in the lungs which play a large role in managing inflammation. Importantly, the diversity of the microbiota in the lungs at birth is fairly low and increases significantly during the first 2 weeks after birth. This shift in microbiota is associated with a shift from CD4^+^FoxP3^+^CD25^+^Helios^+^ Tregs being the primary Treg subset to the emergence of Helios^-^ Tregs that require interaction with PD-L1 (12). While this work supports the premise that the lung microbiota induces regulatory cells in early life, it is also plausible that the Treg populations mediate the development of the microbiota at each stage.

An interesting phenomenon that has gained increasing attention is the gut-lung axis, of which the microbiota and inflammatory signals from each can have a large impact on the other. A recent study determined that maternal-derived γ/δ T cells have an important role in shaping offspring type 2 immune responses in a microbiota-dependent manner (13). Specifically, offspring of γ/δ^-/-^ dams acquired distinct intestinal microbiota which had decreased production of short chain fatty acids (SCFAs), often the end products of bacterial metabolism and a key mediator of inflammatory responses in the neonatal period. This led to increased type 2 inflammation in the lung, demonstrating a microbiota-dependent gut-lung inflammatory axis (13). This work suggests that γ/δ T cells are an important immune component for the selection of gut microbiota that reduce or prevent inflammation in the lung.

The healthy assimilation of respiratory microbiota can be severely disrupted by administration of antibiotics to neonates. In experiments with rhesus macaques, antibiotic administration after birth resulted in delayed maturation of intestinal microbiota, which was associated with reduced hematopoietic cytokines and decreased neutrophil populations. Challenging such animals with *S. pneumoniae* revealed that antibiotics treatment increased inflammatory reactions in the lungs, mainly from neutrophils and AMs (14), suggesting substantially more cross-talk with microbiota than would be predicted by the conventional view of the NIS as incompletely developed. Viewing the NIS as mediating acquisition of a healthy microbiota, such cross-talk might be an expected outcome of the antibiotic’s disruption of the development of the holobiont. Indeed, the NIS of the respiratory tract may require certain signals from resident microbiota to efficiently develop into an AIS. Understanding those signals might be critical to guiding the treatment of neonatal respiratory infections, which currently center on antibiotics that may complicate AIS development in this critical organ.

### 2.3 Gastrointestinal tract

The most diverse and widely appreciated microbiota is that of the gastrointestinal tract (GI). The mammalian GI microbiota is substantially affected by antibodies from mother’s breast colostrum/milk. Despite the protection granted by maternal-derived factors, neonates are highly susceptible to bacterial meningitis, specifically with GBS which typically originates in the GI tract. However, recent research suggests that this susceptibility is not completely dependent on the microbiota, but the structure of the GI tract and immune system (15). The Wnt signaling pathway, which regulates cellular calcium levels, has been recently observed to be age-dependent, not strictly microbiota dependent as previously thought. This pathway in the neonatal gut is increased, leading to decreased cell-cell junction polarization (15). Recent work indicates that postnatal replenishment of enteric glial cells (EGCs) is dependent on the microbiota (16). However, Myd88^-/-^ mice that are defective in signaling pathways involved in detecting microbes lack several EGC markers (16). These observations suggest that the replenishment of EGCs is dependent on both the intestinal microbiota and the Myd88 pathway for detecting them, supporting the view of substantial crosstalk in development. Observation of age-dependent expression of the flagellin receptor TLR5 in the mouse gut epithelium, which mediates REG3γ production, critical for the counter-selection of colonizing flagellated bacteria, further supports this view. Thus, neonatal TLR5 expression influences the composition of the microbiota throughout life and potentially selects for unflagellated commensals and against possible pathogenic flagellated bacteria (17).

In addition to substantial differences in receptors and pathways, the neonatal GI system includes unique T cell populations and functions which can affect microbiota development. A commensal *Propionibacterium* strain, P. UF1 isolated from the gut of newborn mice caused increased numbers of Th17 cells and maintained IL-10^+^ Tregs and protected them from *Listeria monocytogenes.* P. UF1 was also associated with expression of neonatal murine genes that regulate Th17 cell differentiation, suggesting that this intestinal commensal regulates T cell-mediated immunity (18). However, it is also possible that the increase in Th17 cells regulates the populations of P. UF1. Additional work has observed that specific components of the neonatal intestinal microbiota are required for effective protection against pathogens (19). Neonatal mice lacking *Clostridailes* ssp. were unable to prevent colonization by *Salmonella enterica* serovar Typhimurium or *C. rodentium* (19). This suggests that the NIS has co-evolved with commensals such as *Clostridailes* ssp. to allow, and perhaps encourage, their colonization, which then acts as a competitive force to prevent pathogenic colonization.

## 3 Conclusion

We have previously argued that, in addition to protecting against pathogens, a critical role of the mammalian adult immune system is in mediating the complex interactions with the very diverse set of symbionts and commensals that constitute our healthy microbiota (4). The NIS undoubtedly must deal with occasional pathogens, and it is reasonable to consider that this process is greatly complicated by the onslaught of new microorganisms and antigens first encountered after birth, requiring some modulation of response. But it may be myopic to view non-pathogens as simply distracting the NIS from its primary role of repelling pathogens. Viewing the immune system in general as a complex system of microbiota management changes the perspective on the unique challenges of the NIS. The NIS mediates interactions with microorganisms during the transition from near sterile infant in uterus to microbiota-rich healthy holobiont. There may be substantial insight from the broader perspective of the NIS as uniquely evolved to not just survive this onslaught, but to mediate the acquisition of those that can potentially contribute to a healthy holobiont. Hosts as diverse as plants, fungi, algae, insects and cephalopods have evolved complex mechanisms to acquire and maintain their symbionts. Indeed, there is increasing evidence of the many effects our microbiota can have on our health. Considering a, or perhaps the, primary role of the NIS to be mediating the acquisition of a healthy microbiota leads to very different interpretation of its differences to the AIS, focusing on the potential evolutionary advantages of some of these profound differences. We propose that this alternative perspective may generate more questions, hypotheses, and ultimately better understanding of the NIS.

## Data availability statement

The original contributions presented in the study are included in the article/supplementary material. Further inquiries can be directed to the corresponding author.

## Author contributions

CS: Writing – original draft, Writing – review & editing. EH: Writing – original draft, Writing – review & editing.
